# On summary measure analysis of linear trend repeated measures data: performance comparison with two competing methods

**DOI:** 10.1186/1471-2288-12-33

**Published:** 2012-03-22

**Authors:** Mehrdad Vossoughi, SMT Ayatollahi, Mina Towhidi, Farzaneh Ketabchi

**Affiliations:** 1 Department of Biostatistics, Medical School, Shiraz University of Medical Sciences, Shiraz, Iran; 2 Department of Statistics, College of Sciences, Shiraz University, Shiraz, Iran; 3 Department of Physiology, Medical School, Shiraz University of Medical Sciences, Shiraz, Iran

## Abstract

**Background:**

The summary measure approach (SMA) is sometimes the only applicable tool for the analysis of repeated measurements in medical research, especially when the number of measurements is relatively large. This study aimed to describe techniques based on summary measures for the analysis of linear trend repeated measures data and then to compare performances of SMA, linear mixed model (LMM), and unstructured multivariate approach (UMA).

**Methods:**

Practical guidelines based on the least squares regression slope and mean of response over time for each subject were provided to test time, group, and interaction effects. Through Monte Carlo simulation studies, the efficacy of SMA vs. LMM and traditional UMA, under different types of covariance structures, was illustrated. All the methods were also employed to analyze two real data examples.

**Results:**

Based on the simulation and example results, it was found that the SMA completely dominated the traditional UMA and performed convincingly close to the best-fitting LMM in testing all the effects. However, the LMM was not often robust and led to non-sensible results when the covariance structure for errors was misspecified. The results emphasized discarding the UMA which often yielded extremely conservative inferences as to such data.

**Conclusions:**

It was shown that summary measure is a simple, safe and powerful approach in which the loss of efficiency compared to the best-fitting LMM was generally negligible. The SMA is recommended as the first choice to reliably analyze the linear trend data with a moderate to large number of measurements and/or small to moderate sample sizes.

## Background

In many fields of science, repeated measurements of a response variable are taken on each subject over time to assess the changes in response. The cumbersome aspect in analyzing such data is that there are relationships between the measurements in the subject over time. There are two major policies in terms of overcoming or taking the relationships into account.

First, one can reduce the vector of responses of each subject to a single value by a descriptive statistic and apply standard univariate approaches to test the effects related to the corresponding summary measure. The use of the summary measure approach (SMA) was suggested by Wishart [1] for the first time. Several strategies based on the least squares regression slope and mean of response over time were recommended to evaluate the differences between the groups [2-6]. Moreover, the utility of Kendall's *τ_b _*as a summary measure of within-subjects trend in psychiatric longitudinal studies, where the key assumptions of parametric methods are not held, was investigated [7,8].

Second, one can use methods which take the covariances between the measurements into account. Two common and traditional approaches for normally distributed responses are repeated measures ANOVA and MANOVA. In order to avoid inflating type I error rate, the denominator degrees of freedom of the *F *statistics in the repeated measures ANOVA approach should be adjusted under departures from a restrictive assumption on covariance structures, namely sphericity. But there is no obvious advantage in using the adjusted *F *tests against the multivariate tests, and generally the adjustments should be avoided [9,10]. In contrast, the repeated measures MANOVA approach makes no assumption regarding covariance structure and hence, it is sometimes known as unstructured multivariate approach (UMA). The only key advantage of the repeated measures ANOVA approach over the UMA is that it can still be implemented in the case where the number of measurements is greater than the sample size.

The linear mixed model (LMM) is more advanced and flexible since it allows dealing with subjects which have incomplete measurements and are unequally spaced in the time period. But the performance of the LMM in testing the effects is highly dependent on the choice of appropriate covariance structure for errors [11,12]. On the other hand, the choice of a parsimonious covariance structure in a small sample design can lead to more efficient inferences concerning the fixed-effects parameters. This aspect makes it inconvenient and unreliable, especially for those who are not familiar with the fundamental principles of mixed models.

Although SMA is a simple, robust and sometimes only applicable tool for the analysis of repeated measures studies, there exists no obvious performance comparison on using the SMA vs. other competitors. Moreover, the application of the SMA has been mostly based on using one summary statistic to assess only the total group difference.

The present study includes repeated measures data in which the pattern of the response profile can be described by a linear trend and the responses measured in a continuous scale. The main objectives of this study are:

a) To describe techniques to test time (within-subjects), group (between-subjects) and group × time interaction effects on the basis of two common summary measures, i.e. least square regression slope and mean of response over time.

b) To compare the performance of the SMA, LMM and UMA in the analysis of simulated data from a LMM framework under different types of covariance structures. The approach is also illustrated and compared with the competitors using two real data sets.

In our simulations, there is a focus on situations where the LMM may provide extremely unsatisfactory performance such as misspecification of the covariance structure for errors, small and moderate sample sizes, and relatively a large number of measurements.

## Methods

### Unstructured multivariate approach (UMA)

The UMA handles the measurements in the subject as a vector of multivariate responses and treats time points as levels of a qualitative factor with no order. This approach is restricted in equally spaced time points, balanced data with complete measurements and also assumes the homogeneity of covariance matrices in all the *k *groups.

Let $Y_{ih}={\left(Y_{i1h},\ldots ,Y_{imh}\right)}^{T}$ denote the vector of *m *responses from the *i*th subject in group *h *for *i *= 1,...,*n_h_, h *= 1,...,*k*. It is assumed that the response vectors, ***Y_ih_***, are independent and have multivariate normal distribution with mean $\mu_{h}={\left(\mu_{1h},...,\mu_{mh}\right)}^{T}$ and common covariance matrix ***Σ***. The total mean vector is also defined as ${\bar{\mu }}_{.}=1/k\sum_{h=1}^{k}\mu_{h}$. If there is no additional covariate, one can use a profile model as

(1) $$Y_{ih}=\mu_{h}+\varepsilon_{ih},$$

where the vector $\varepsilon_{ih}={\left(\varepsilon_{i1h},...,\varepsilon_{imh}\right)}^{T}$ is the vector of error for the *i*th subject in group *h*.

The primary hypothesis interest in a profile analysis is the parallelism of the *k *groups' profiles or no group × time interaction effect. The hypothesis can be constructed as *H_0_*: ***C μ_1 _***= ... = ***C μ_k _***for an appropriate transformation matrix ***C ***with rank *m-*1. If the test of interaction is not significant, the tests of the main effects are not confounded. In order to compute any MANOVA-type test statistics such as Wilk's lambda (*Λ*), the condition *N*-*k *>*m-*1 is necessary, where *N *is the total number of subjects. Otherwise, the estimated covariance matrix of the transformed responses would not be non-singular and positive-definite. To test time effect, one can investigate the equality of the *m *elements of the total mean vector (${\bar{\mu }}_{.}$) using one-sample Hotelling's *T^2 ^*test on the *m*-1 differences between adjacent measurements from each subject. Here, the same strategy as the SMA is utilized to test group effect, as it is often more efficient than MANOVA-type tests to compare the groups' mean vectors.

### Linear mixed model (LMM)

Let $Y_{i}={\left(Y_{i1},...,Y_{im}\right)}^{T}$ denote the *m_i _*× 1 vector of responses from the *i*th subject for *i *= 1,...,*N*, where *N *is the total number of subjects. In contrast to the UMA, the subjects may have different measuring time points and be unbalanced in terms of the number of measurements. The general form of the LMM is

(2) $$Y_{i}=X_{i}^{T}\beta +Z_{i}^{T}b_{i}+\varepsilon_{i},$$

where $X_{i}^{T}$ is an *m_i _*× *p *fixed-effects design matrix for the *i*th subject, ***β ***is a *p *× 1 vector of fixed-effects parameters for the population, ***b_i _***is a *q *× 1 vector of random effects for the *i*th subject, $Z_{i}^{T}$ is an *m_i _*× *q *random-effects design matrix for the *i*th subject with *q *≤ *p*, and ***ε_i _***is an *m_i _*× 1 vector of within-subject errors. The random-effects vectors, ***b_i_***, are assumed to be independent and to have a multivariate normal distribution with mean zero and covariance matrix ***G_i_***, and the error vectors, ***ε_i_***, are assumed to be independent and to have a multivariate normal distribution with mean zero and covariance matrix ***R_i_***. In addition, it is also assumed that ***b_i _***and ***ε_i _***are independent of one another. The LMM defines the covariances of the measurements in the subject by the covariances of the random effects (***G_i_***) and the covariances of the errors (***R_i_***). We used the estimators based on the restricted maximum likelihood (REML) method to construct the *F *statistics of the hypotheses since, in general, it yields less biased estimates of the variance components than those of maximum likelihood (ML) approach and avoids inflating type I error rates [12,13].

### The summary measure approach (SMA)

In this section, we describe how to apply the least squares regression slope and mean of response over time for each subject to test the effects of time, group and group × time interaction in repeated measures studies.

The slope of least squares regression line was applied to summarize the relationship between response and time for each subject or within-subjects effect. If the pattern of individual profiles is linear or at least monotonic, the slopes can appropriately summarize the rate of change of response over time in the subjects. For repeated measures designs, the primary hypothesis is to test whether the pattern of change over time is the same across the *k *groups or no group × time interaction effect. Under the assumption of no interaction effect, the slopes in the *k *groups should not be significantly different. For this purpose, once the slopes are obtained for each subject, the ordinary *k *sample tests such as one-way ANOVA *F *or Kruskal-Wallis (for *k *> 2) and Student's *t *or Wilcoxon-Mann-Whitney (for *k *= 2) can be employed to assess the equality of the slopes in the groups. If the test of interaction is not significant, one would be interested in assessing the main effects.

The hypothesis of no time (within-subjects) effect states that all the *m *elements of the total mean vector (${\bar{\mu }}_{.}$) are identical. Under this assumption, the overall mean of the slopes in the population must be zero. To test this hypothesis, one-sample *t *test can be applied to the sample slopes to assess the departure of mean slopes from zero.

For testing group (between-subjects) effect, the mean of measurements over time for each subject is used as a summary measure. By analogy with the interaction effect case, the ordinary *k *sample tests are applied, but this time, to assess the equality of the individual means in the groups.

Permutation procedure can also be employed to assess the interaction and group effects where the constructive assumptions of the standard tests are not held or cannot be reasonably checked due to small sample sizes in the groups.

### Simulation study

For the purpose of data simulation, a simple linear trend mixed model with a random coefficient only for the intercept and a two category grouping variable was considered. The model can be expressed as

(3) $$Y_{ij}=\beta_{0}+\beta_{1}X_{i}+\beta_{2}t_{ij}+\beta_{3}\left(t_{ij}\times X_{i}\right)+b_{0i}+\varepsilon_{ij},$$

where *Y_ij _*is the *j*th measurement from *i*th subject and *X*_i _is a grouping variable with the values 0 and 1, for *i *= 1,...,*N *and *j *= 1,...,*m_i_*.

Linear trend mixed model data was generated based on the model (3) with the same measuring time points *t_ij _*= *t_j _*= 2*j *for all the subjects, *m_i _*= *m *= 5, 10, and 20 measurements and *β_0 _*= 2, in which the random effects, *b_0i_*, were assumed to be independently normally distributed with mean zero and standard deviation 0.25.

Since hypothesis testing effects related to within-subjects effect is highly dependent on the number of measurements, the values of *β_2 _*and *β_3 _*are adjusted with respect to the *m *values. Different combinations of *β_1_, β_2 _*and *β_3 _*were constructed to compute the empirical type I error rates and powers for testing the three effects.

We considered the following three covariance structures for errors to generate artificial data and fit the LMMs:

• Simple or independent (IND): ***R_i _***= *σ^2^**I***, where ***I ***is an *m *× *m *identity matrix.

• First-order autoregressive (AR1) with *ρ *= 0.7: ***R_i _***= *σ^2^**H***, where ***H ***= [*h_jj'_*] is an *m *× *m *matrix with *h_jj' _*= *ρ*^|*j-j'*| ^for all *j *and *j'*.

• Unstructured (UNS): ***R_i _***= [*r_jj'_*] is an *m *× *m *covariance matrix with arbitrary structure.

For simplicity, we defined the true structures as those which were used to generate data and the working structures as those which were used to fit the model. In all the cases, it was assumed that the errors were normally distributed with zero mean and in the cases of IND and AR1, the error variances were fixed over time and equal to *σ^2 ^*= 0.5.

1000 sample data sets were generated for *n_1 _*= *n_2 _*= *n_3 _*= 5, 10, 30 and 50 subjects under various choices of the above circumstances.

We have used free statistical software environment R to generate the artificial datasets and fit all of the approaches presented in the method section.

## Results

### Simulation results

#### Within-subject (time) and within-by-between-subjects (interaction) effects

Tables 1 and 2 display the empirical type I error rates and powers of the tests of time and interaction effects for various covariance structures, respectively. The first rows in each part, where *β_1 _*= *β_2 _*= 0 (*β_3 _*= 0), display the empirical type I error rates and the rows corresponding to *β_1 _*> 0 and *β_2 _*> 0 (*β_3 _*> 0) show the empirical powers in testing time (interaction) effect. Because of the similarities between the results of testing time and interaction effects, we combined the results in this section in which the following report is right for both effects.

**Table 1:** Type I error rates and powers for testing within-subjects (time) effect where rows with *β_1 _*= 0 and *β_2 _*= 0 give the type I error rates, and the other rows are powers

			True covariance structure														
			**IND**					**AR1**					**UNS**				

			**LMM working covariance**					**LMM working covariance**					**LMM working covariance**				
											
**(*m, n*)**	***β_1_***	***β_2_***	**IND**	**AR1**	**UNS**	**SMA**	**UMA**	**IND**	**AR1**	**UNS**	**SMA**	**UMA**	**IND**	**AR1**	**UNS**	**SMA**	**UMA**

(5,5)	0	0	0.052	0.071	0.230	0.059	0.052	0.188	0.079	0.230	0.050	0.043	0.045	0.075	0.252	0.059	0.041
	0.25	0.020	0.097	0.118	0.258	0.086	0.064	0.264	0.129	0.288	0.081	0.064	0.120	0.169	0.352	0.112	0.065
	0.35	0.040	0.197	0.216	0.373	0.181	0.080	0.418	0.251	0.418	0.168	0.070	0.287	0.378	0.590	0.289	0.140
(5,10)	0	0	0.052	0.070	0.100	0.053	0.046	0.165	0.072	0.109	0.047	0.055	0.035	0.060	0.101	0.040	0.042
	0.25	0.020	0.114	0.133	0.165	0.107	0.057	0.317	0.146	0.194	0.115	0.083	0.159	0.212	0.315	0.171	0.124
	0.35	0.040	0.352	0.370	0.429	0.338	0.188	0.577	0.393	0.443	0.315	0.183	0.565	0.634	0.765	0.565	0.428
(5,30)	0	0	0.044	0.065	0.069	0.043	0.051	0.177	0.056	0.064	0.053	0.051	0.051	0.065	0.065	0.058	0.053
	0.25	0.020	0.305	0.320	0.331	0.296	0.163	0.530	0.316	0.335	0.287	0.171	0.458	0.513	0.629	0.466	0.393
	0.35	0.040	0.785	0.792	0.809	0.772	0.532	0.910	0.809	0.810	0.775	0.576	0.963	0.975	0.995	0.958	0.965
(5,50)	0	0	0.049	0.054	0.058	0.048	0.050	0.173	0.059	0.050	0.057	0.047	0.048	0.069	0.045	0.041	0.045
	0.25	0.020	0.437	0.443	0.450	0.431	0.243	0.681	0.480	0.485	0.424	0.262	0.686	0.737	0.783	0.715	0.658
	0.35	0.040	0.940	0.939	0.941	0.941	0.810	0.988	0.960	0.972	0.950	0.831	0.998	0.999	1.000	0.999	1.000
(10,5)	0	0	0.049	0.071	--*	0.048	--†	0.277	0.070	--*	0.058	--†	0.047	0.060	--*	0.048	--†
	0.25	0.010	0.158	0.179	--*	0.148	--†	0.366	0.105	--*	0.070	--†	0.176	0.194	--*	0.156	--†
	0.35	0.020	0.360	0.371	--*	0.286	--†	0.519	0.256	--*	0.157	--†	0.492	0.488	--*	0.409	--†
(10,10)	0	0	0.065	0.078	0.265	0.059	0.043	0.290	0.063	0.277	0.055	0.057	0.040	0.043	0.279	0.043	0.059
	0.25	0.010	0.200	0.217	0.453	0.189	0.078	0.423	0.134	0.385	0.121	0.065	0.271	0.277	0.500	0.266	0.090
	0.35	0.020	0.636	0.652	0.772	0.580	0.152	0.689	0.401	0.600	0.327	0.107	0.815	0.814	0.879	0.786	0.279
(10,30)	0	0	0.063	0.076	0.086	0.062	0.039	0.268	0.053	0.093	0.049	0.039	0.040	0.043	0.092	0.038	0.045
	0.25	0.010	0.507	0.517	0.570	0.488	0.197	0.621	0.310	0.385	0.278	0.136	0.709	0.714	0.898	0.697	0.341
	0.35	0.020	0.979	0.979	1.000	0.974	0.715	0.949	0.824	0.879	0.764	0.420	1.000	1.000	1.000	0.999	0.959
(10,50)	0	0	0.055	0.058	0.067	0.054	0.049	0.257	0.051	0.061	0.049	0.041	0.048	0.048	0.067	0.056	0.054
	0.25	0.010	0.716	0.718	0.725	0.712	0.310	0.752	0.484	0.500	0.428	0.204	0.893	0.896	0.940	0.893	0.603
	0.35	0.020	0.999	0.996	1.000	0.999	0.958	0.993	0.961	0.970	0.941	0.684	1.000	1.000	1.000	1.000	1.000
(20,5)	0	0	0.062	0.070	--*	0.054	--†	0.369	0.066	--*	0.054	--†	0.049	0.050	--*	0.050	--†
	0.25	0.005	0.203	0.218	--*	0.164	--†	0.462	0.098	--*	0.076	--†	0.235	0.241	--*	0.242	--†
	0.35	0.010	0.619	0.621	--*	0.529	--†	0.606	0.265	--*	0.1208	--†	0.700	0.711	--*	0.706	--†
(20,10)	0	0	0.065	0.069	--*	0.058	--†	0.378	0.056	--*	0.046	--†	0.040	0.040	--*	0.040	--†
	0.25	0.005	0.394	0.394	--*	0.380	--†	0.530	0.171	--*	0.143	--†	0.469	0.472	--*	0.481	--†
	0.35	0.010	0.901	0.902	--*	0.870	--†	0.790	0.472	--*	0.394	--†	0.960	0.961	--*	0.961	--†
(20,30)	0	0	0.047	0.050	0.173	0.049	0.057	0.369	0.051	0.179	0.045	0.049	0.049	0.049	0.178	0.051	0.061
	0.25	0.005	0.794	0.794	0.888	0.789	0.223	0.705	0.355	0.435	0.327	0.111	0.920	0.920	1.000	0.918	0.392
	0.35	0.010	1.000	1.000	1.000	1.000	0.857	0.977	0.889	0.940	0.832	0.293	1.000	1.000	1.000	1.000	0.985
(20,50)	0	0	0.047	0.049	0.102	0.045	0.043	0.360	0.045	0.098	0.034	0.039	0.046	0.047	0.104	0.049	0.046
	0.25	0.005	0.979	0.976	1.000	0.942	0.441	0.849	0.567	0.628	0.479	0.154	1.000	1.000	1.000	0.993	0.775
	0.35	0.010	1.000	1.000	1.000	1.000	0.996	1.000	0.991	1.000	0.975	0.628	1.000	1.000	1.000	1.000	1.000

*: Since *m *was large relative to the sample size (*N *= 2*n*), modeling with large number of parameters was discarded.

†: Since *N*-*k *<*m*-1, the UMA could not be conducted

**Table 2:** Type I error rates and powers for testing group × time interaction effect where rows with *β_3 _*= 0 give the type I error rates, and the other rows are powers

		True covariance structure														
		**IND**					**AR1**					**UNS**				

		**LMM working covariance**					**LMM working covariance**					**LMM working covariance**				
										
**(*m, n*)**	***β_3_***	**IND**	**AR1**	**UNS**	**SMA**	**UMA**	**IND**	**AR1**	**UNS**	**SMA**	**UMA**	**IND**	**AR1**	**UNS**	**SMA**	**UMA**

(5,5)	0	0.057	0.065	0.222	0.043	0.043	0.192	0.079	0.270	0.047	0.055	0.044	0.074	0.251	0.042	0.068
	0.040	0.074	0.087	0.260	0.065	0.052	0.240	0.126	0.276	0.076	0.061	0.095	0.160	0.358	0.099	0.086
	0.080	0.209	0.232	0.399	0.167	0.095	0.424	0.242	0.391	0.141	0.071	0.278	0.361	0.589	0.247	0.114
(5,10)	0	0.050	0.062	0.099	0.049	0.059	0.185	0.064	0.100	0.047	0.050	0.040	0.060	0.100	0.044	0.042
	0.040	0.128	0.135	0.196	0.111	0.090	0.381	0.136	0.202	0.099	0.076	0.155	0.202	0.316	0.161	0.117
	0.080	0.351	0.362	0.412	0.329	0.166	0.776	0.405	0.478	0.317	0.166	0.545	0.621	0.766	0.569	0.442
(5,30)	0	0.049	0.054	0.062	0.050	0.056	0.188	0.055	0.057	0.055	0.048	0.045	0.064	0.056	0.056	0.062
	0.040	0.295	0.305	0.316	0.285	0.171	0.501	0.297	0.302	0.263	0.156	0.465	0.532	0.637	0.488	0.401
	0.080	0.807	0.810	0.823	0.779	0.522	0.973	0.828	0.840	0.786	0.580	0.968	0.980	0.995	0.971	0.976
(5,50)	0	0.038	0.039	0.045	0.041	0.039	0.176	0.059	0.058	0.054	0.056	0.039	0.062	0.049	0.048	0.040
	0.040	0.421	0.425	0.429	0.414	0.235	0.685	0.445	0.446	0.393	0.239	0.685	0.745	0.780	0.717	0.674
	0.080	0.952	0.952	0.954	0.948	0.824	1.000	0.954	0.957	0.946	0.838	0.998	0.998	1.000	0.998	0.998
(10,5)	0	0.059	0.065	--*	0.052	--†	0.300	0.072	--*	0.039	--†	0.050	0.059	--*	0.042	--†
	0.020	0.129	0.138	--*	0.100	--†	0.412	0.123	--*	0.071	--†	0.172	0.189	--*	0.159	--†
	0.040	0.362	0.371	--*	0.280	--†	0.540	0.248	--*	0.146	--†	0.545	0.545	--*	0.418	--†
(10,10)	0	0.050	0.053	0.275	0.055	0.045	0.309	0.045	0.241	0.040	0.039	0.044	0.044	0.281	0.049	0.044
	0.020	0.177	0.177	0.400	0.184	0.078	0.453	0.162	0.375	0.129	0.066	0.284	0.289	0.493	0.275	0.113
	0.040	0.323	0.326	0.641	0.576	0.178	0.707	0.391	0.616	0.299	0.097	0.828	0.825	0.840	0.772	0.254
(10,30)	0	0.050	0.051	0.097	0.047	0.048	0.278	0.051	0.088	0.060	0.057	0.059	0.059	0.095	0.053	0.049
	0.020	0.502	0.502	0.587	0.494	0.191	0.596	0.297	0.344	0.259	0.112	0.660	0.660	0.774	0.673	0.320
	0.040	0.977	0.972	1.000	0.977	0.725	0.980	0.802	0.866	0.747	0.370	1.000	1.000	1.000	0.997	0.943
(10,50)	0	0.052	0.052	0.075	0.049	0.047	0.283	0.058	0.072	0.049	0.065	0.050	0.052	0.078	0.048	0.049
	0.020	0.698	0.696	0.708	0.686	0.351	0.762	0.460	0.471	0.399	0.180	0.876	0.877	0.909	0.887	0.600
	0.040	0.998	0.998	1.000	0.998	0.949	1.000	0.966	0.970	0.938	0.686	1.000	1.000	1.000	1.000	1.000
(20,5)	0	0.049	0.055	--*	0.044	--†	0.340	0.052	--*	0.048	--†	0.050	0.054	--*	0.054	--†
	0.010	0.208	0.217	--*	0.157	--†	0.436	0.119	--*	0.083	--†	0.210	0.218	--*	0.211	--†
	0.020	0.614	0.619	--*	0.489	--†	0.647	0.288	--*	0.200	--†	0.641	0.639	--*	0.655	--†
(20,10)	0	0.050	0.057	--*	0.051	--†	0.329	0.053	--*	0.043	--†	0.049	0.053	--*	0.054	--†
	0.010	0.361	0.361	--*	0.316	--†	0.788	0.154	--*	0.122	--†	0.446	0.448	--*	0.445	--†
	0.020	0.911	0.908	--*	0.863	--†	0.803	0.460	--*	0.382	--†	0.972	0.971	--*	0.955	--†
(20,30)	0	0.057	0.055	0.178	0.063	0.065	0.350	0.048	0.169	0.044	0.041	0.052	0.052	0.181	0.048	0.050
	0.010	0.808	0.808	0.928	0.795	0.258	0.736	0.384	0.444	0.340	0.101	0.914	0.914	1.000	0.927	0.399
	0.020	1.000	1.000	1.000	1.000	0.871	1.000	0.914	1.000	0.855	0.293	1.000	1.000	1.000	1.000	0.987
(20,50)	0	0.049	0.046	0.095	0.046	0.049	0.346	0.042	0.089	0.042	0.051	0.049	0.050	0.104	0.049	0.052
	0.010	0.935	0.932	0.986	0.926	0.442	0.833	0.525	0.550	0.482	0.154	0.990	0.992	1.000	0.992	0.715
	0.020	1.000	1.000	1.000	1.000	0.997	1.000	0.986	1.000	0.969	0.597	1.000	1.000	1.000	1.000	1.000

*: Since *m *was large relative to the sample size (*N *= 2*n*), modeling with large number of parameters was discarded.

†: Since *N*-*k *<*m*-1, the UMA could not be conducted

First, the three approaches are compared under the IND and AR1 as true structures. As illustrated in both Tables 1 and 2, empirical type I error rates of the SMA and UMA were always close to (and often smaller than) the nominal significance level (%5). However, the LMM in testing both effects displayed notably larger values for the IND working structure under the AR1 as true structure and more generally for the UNS working structure under the two true structures. Unfortunately, the inflation of type I error rates for the IND working structure under the AR1 true structure tended to be fixed as *n *increased. As misleading results, because of not preserving the type I error rates, the empirical powers of the LMM in these cases were notably greater than those obtained by the other approaches. In summary, the empirical powers of the SMA were notably greater than those of the UMA and were often close to the corresponding values of the best-fitting LMM. It is also worth mentioning that while the powers of the SMA and LMM tend to be 1 for some larger values of *n*, the values of the UMA have evident departures from them such as *n *= 50 with *m *= 5, 10, 20 and somewhat *n *= 30 with *m *= 10, 20.

Next, we consider the simulation results for the UNS as true covariance structure in testing time and interaction effects. The LMMs with the IND and AR1 working structures preserved the type I error rates again. Interestingly, like the IND and AR1 true structures, the empirical type I error rates of the LMM for the UNS working structure were not preserved for smaller and larger values of *n *and *m*, respectively. It is worthwhile to note that the empirical type I error rates of the LMM were relatively comparable to the corresponding values of the other approaches only for larger values of n accompanied by smaller values of *m; n *= 30 and 50 with *m *= 5, and somewhat, *n *= 50 with *m *= 10 in both Tables. However, the empirical powers of the SMA and all the LMMs were similar under such circumstances. Only in the case of *m *= 5 with *n *= 30, 50 under the UNS true structure, the UMA was comparable with the SMA in testing both effects.

#### Between-subjects (group) effect

The empirical type I error rates and powers of the test of group effect are displayed in Table 3 in which the empirical type I error rates are the values of the first rows where *β_1 _*= *β_2 _*= 0 and the other rows, where *β_1 _*> 0 and *β_2 _*> 0, display the empirical powers. Again, it should be noted that both the SMA and UMA use the same strategy to test group effect. Hence, Table 3 only reported the results for the SMA and LMM.

**Table 3:** Type I error rates and powers for testing between-subjects (group) effect where rows with *β_1 _*= 0 and *β_2 _*= 0 give the type I error rates, and the other rows are powers

			True covariance structure											
			**IND**				**AR1**				**UNS**			

			**LMM working covariance**				**LMM working covariance**				**LMM working covariance**			
								
**(*m, n*)**	***β_1_***	***β_2_***	**IND**	**AR1**	**UNS**	**SMA(UMA)**	**IND**	**AR1**	**UNS**	**SMA(UMA)**	**IND**	**AR1**	**UNS**	**SMA(UMA)**

(5,5)	0	0	0.031	0.038	0.231	0.042	0.049	0.037	0.189	0.040	0.049	0.048	0.217	0.043
	0.25	0.020	0.132	0.142	0.336	0.142	0.085	0.071	0.211	0.078	0.103	0.102	0.269	0.095
	0.35	0.040	0.211	0.207	0.409	0.213	0.138	0.126	0.273	0.123	0.146	0.145	0.313	0.124
(5,10)	0	0	0.052	0.056	0.082	0.054	0.058	0.045	0.082	0.055	0.053	0.052	0.088	0.051
	0.25	0.020	0.253	0.260	0.291	0.257	0.144	0.146	0.177	0.143	0.149	0.151	0.188	0.144
	0.35	0.040	0.451	0.448	0.473	0.450	0.239	0.223	0.249	0.234	0.259	0.259	0.298	0.255
(5,30)	0	0	0.044	0.046	0.052	0.044	0.047	0.051	0.058	0.046	0.053	0.055	0.061	0.053
	0.25	0.020	0.673	0.673	0.682	0.672	0.332	0.336	0.346	0.331	0.386	0.382	0.394	0.386
	0.35	0.040	0.923	0.922	0.939	0.922	0.578	0.581	0.590	0.578	0.621	0.619	0.622	0.620
(5,50)	0	0	0.049	0.049	0.049	0.046	0.050	0.054	0.051	0.050	0.047	0.046	0.045	0.047
	0.25	0.020	0.886	0.884	0.890	0.886	0.509	0.580	0.577	0.509	0.569	0.570	0.534	0.566
	0.35	0.040	0.986	0.986	0.990	0.986	0.800	0.804	0.806	0.800	0.847	0.847	0.839	0.852
(10,5)	0	0	0.039	0.043	--*	0.034	0.034	0.030	--*	0.038	0.037	0.038	--*	0.035
	0.25	0.010	0.175	0.178	--*	0.174	0.079	0.079	--*	0.089	0.107	0.106	--*	0.097
	0.35	0.020	0.295	0.296	--*	0.286	0.143	0.144	--*	0.153	0.141	0.143	--*	0.125
(10,10)	0	0	0.050	0.052	0.244	0.050	0.065	0.047	0.237	0.063	0.047	0.048	0.259	0.045
	0.25	0.010	0.330	0.330	0.541	0.328	0.173	0.175	0.348	0.172	0.185	0.185	0.393	0.182
	0.35	0.020	0.574	0.570	0.709	0.573	0.307	0.300	0.470	0.302	0.302	0.300	0.451	0.298
(10,30)	0	0	0.043	0.049	0.058	0.043	0.050	0.048	0.058	0.050	0.049	0.050	0.057	0.049
	0.25	0.010	0.822	0.822	0.840	0.822	0.454	0.453	0.462	0.454	0.465	0.467	0.480	0.467
	0.35	0.020	0.979	0.971	0.986	0.979	0.726	0.762	0.769	0.726	0.733	0.731	0.743	0.733
(10,50)	0	0	0.045	0.045	0.050	0.045	0.050	0.042	0.050	0.050	0.049	0.049	0.050	0.050
	0.25	0.010	0.952	0.951	0.954	0.952	0.671	0.695	0.671	0.671	0.671	0.671	0.670	0.675
	0.35	0.020	1.000	1.000	1.000	1.000	0.906	0.934	0.935	0.906	0.909	0.909	0.916	0.911
(20,5)	0	0	0.053	0.057	--*	0.043	0.052	0.040	--*	0.049	0.048	0.049	--*	0.038
	0.25	0.005	0.214	0.221	--*	0.204	0.120	0.107	--*	0.103	0.100	0.101	--*	0.091
	0.35	0.010	0.399	0.403	--*	0.369	0.203	0.193	--*	0.185	0.206	0.206	--*	0.189
(20,10)	0	0	0.050	0.050	--*	0.048	0.046	0.046	--*	0.045	0.050	0.053	--*	0.057
	0.25	0.005	0.455	0.455	--*	0.451	0.238	0.237	--*	0.238	0.237	0.239	--*	0.236
	0.35	0.010	0.716	0.714	--*	0.705	0.387	0.392	--*	0.383	0.360	0.359	--*	0.368
(20,30)	0	0	0.055	0.056	0.103	0.055	0.056	0.066	0.097	0.060	0.049	0.049	0.110	0.048
	0.25	0.005	0.899	0.899	0.942	0.899	0.595	0.611	0.646	0.597	0.580	0.580	0.619	0.583
	0.35	0.010	0.993	0.993	1.000	0.993	0.873	0.890	0.917	0.873	0.830	0.832	0.881	0.844
(20,50)	0	0	0.046	0.047	0.079	0.045	0.042	0.042	0.079	0.047	0.049	0.049	0.082	0.050
	0.25	0.005	0.992	0.990	1.000	0.990	0.831	0.855	0.881	0.831	0.781	0.780	0.834	0.796
	0.35	0.010	1.000	1.000	1.000	1.000	0.960	0.979	1.000	0.977	0.999	0.998	1.000	1.000

*: Since *m *was large relative to the sample size (*N *= 2*n*), modeling with large number of parameters was discarded

First, the simulation results for testing the group effect are considered under the IND and AR1 as true covariance structures. Except for the UNS working structure, both SMA (UMA) and LMM often obtained the same empirical type I error rates close to the nominal significance level. Contrary to what we obtained for the two other effects, the LMM preserved the type I error rates for the IND working structure under the AR1 as true structure. The LMM with the UNS working structure tended to have obviously larger empirical type I error rates than the SMA (UMA). The empirical powers of the SMA and LMM were absolutely similar in both IND and AR1 as true structures when the type I error rates were preserved by the LMM.

Now, we consider the results for the UNS as true covariance structure. The LMM with the UNS working structure yielded the preserved type I error rates only for larger values of *n *accompanied by smaller values of *m *such as *n *= 30, 50 with *m *= 5, 10. However, the LMM preserved type I error rates for the IND and AR1 working structures. In these comparable circumstances, the differences in the powers between the SMA and all the LMMs were negligible.

### Illustrative examples

#### Example 1: Pituitary-pteryomaxillary distance data

The first example is a small data set on a facial distance previously published by Potthoff and Roy [14] conducted at the University of North Carolina Dental School. The distance (mm) from the centre of the pituitary gland to the pteryomaxillary fissure was measured at age 8, 10, 12, and 14 in two groups of children (11 girls and 16 boys). The data set has also been analyzed by several analytic methods [12,15].

**Figure 1:**
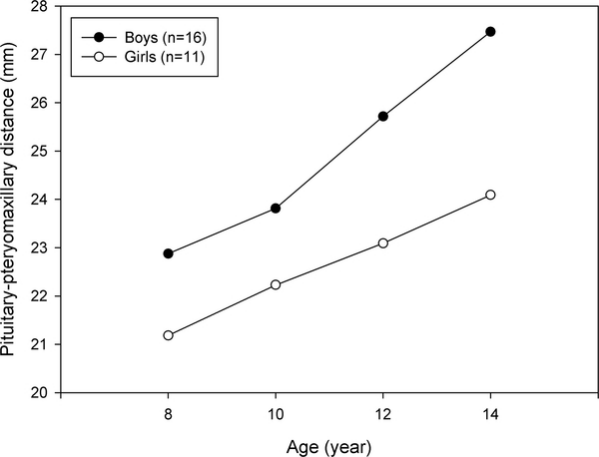
**Mean profiles of pituitary-pteryomaxillary distances**.

Figure 1 displays the mean profiles in boys and girls and indicates a departure from the parallelism hypothesis. In general, boys tend to have larger pituitary-pteryomaxillary distances and a faster growth rate than girls. In addition, the distances increase over age points in both groups of children.

Given model (3), three models were fitted with IND, AR1, and UNS covariance structures. Random intercept and slope models with the three covariance structures were also employed. Table 4 reports the results for the six LMMs, UMA and SMA, as well as Akaike's information criterion (AIC) and Bayesian information criterion (BIC) as two model selection indices for the LMMs. The model with the smallest criterion provides the best fit to data. Based on both foregoing criteria, model 1 with IND covariance structure was preferred. It is worth mentioning that a random intercept model with IND covariance structure for errors yields a compound symmetry covariance structure between the responses.

**Table 4:** Pituitary-pteryomaxillary distances data: summary of test results

			Effect				
					
Method	no	Structure	Group	Time	Group × Time	AIC	BIC
LMM with Random intercept	1	IND	0.005	< 0.001	0.014	445.76	461.62
	
	2	AR1	0.006	< 0.001	0.012	447.71	466.22
	
	3	UNS	0.007	< 0.001	0.009	450.17	481.90

LMM with Random intercept and slope	4	IND	0.009	< 0.001	0.026	448.58	469.74
	
	5	AR1	0.015	< 0.001	0.021	446.81	470.61
	
	6	UNS	0.010	< 0.001	0.009	454.12	491.14

SMA	7	-	0.005	< 0.001	0.019	-	-

UMA	8	-	0.005	< 0.001	0.070	-	-

All the LMMs and the SMA showed a significant interaction effect at the 5% significance level. These results indicated that the growth pattern in boys was faster than that in girls. Although one could not reject the hypothesis of no interaction effect by the UMA, there was some evidence that the profiles in Figure 1 were not parallel.

All the approaches yielded significant results for the two main effects on the facial growth measurements of children. Based on these results, we accept that boys have larger facial distances than girls and the facial distances increase over age in the two groups of children.

#### Example 2: Change in lung NO metabolites level data

The second example is an animal experimental study which is about the effects of hypercapnia with or without acidosis on NO production in the isolated ventilated-perfused rabbit lung by assessment of the NO metabolites (nitrite and nitrate) concentration released into the perfusate. The study was conducted at Justus-Liebig-University, Giessen. The NO metabolites concentration (nmol/min) was measured at time point 0, 5, 10, 15, 30, 45, ..., and 180 minutes in three groups of normoxic normocapnia (NX-NC, *n *= 7), normoxic hypercapnia with acidosis (NX-HCA, *n *= 4) and normoxic hypercapnia with normal pH level (NX-HCN, *n *= 6). Since there were some variations between the baseline measurements, values were given as changes from the baseline. There were six samples (lungs) with incomplete measurements.

**Figure 2:**
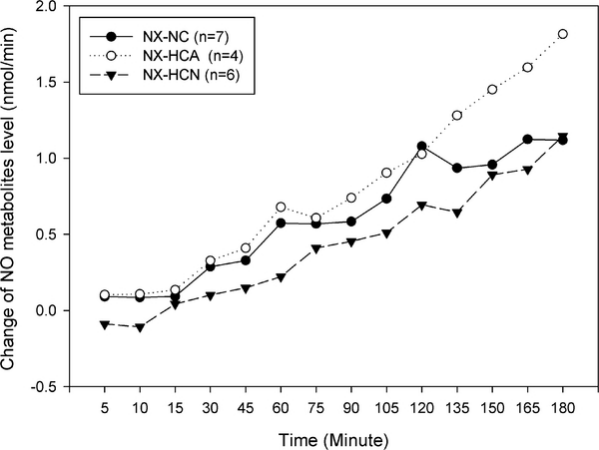
**Mean profiles of change in NO metabolites level**.

Figure 2 displays the mean profiles of change in NO metabolites level data over time for the three groups. The mean profiles increase over time points in all of the groups. However, it is not expected that the patterns of change in NO metabolites level and the overall means will differ between the three conditions.

In this data set, the UMA could not be conducted, because the number of measurements (*m *= 14) was larger than that of the samples with complete measurements (11 lungs) during the period of study. On the other hand, the UMA and repeated measures ANOVA approaches are not able to handle the experimental units with missing observations.

Table 5 displays the results for the LMM with random intercept, random intercept and slope and also the SMA. Note that AIC prefers random intercept and slope model with UNS, IND and AR1 covariance structures, models 6, 4 and 5, respectively, whereas random intercept and slope model with IND and AR1 covariance structures are to be preferred based on BIC, models 4 and 5, respectively. The reason is that a heavier penalty in the calculation of BIC than AIC was imposed when the number of parameters in the model increased. Since there were a limited number of lungs and a large number of measurements, the danger of over-fitting increases. In these cases, it is more reasonable to rely on BIC to select the best parsimonious model. Note that model 6 has larger parameters (*d *= 112) than model 4 (*d *= 8) which must be estimated.

**Table 5:** Change in NO metabolites level data: summary of test results

			Effect				
					
Method	no	Structure	Group	Time	Group × Time	AIC	BIC
LMM with Random intercept	1	IND	0.264	< 0.001	< 0.001	121.38	148.31
	
	2	AR1	0.248	< 0.001	0.026	58.77	89.06
	
	3	UNS	0.175	< 0.001	< 0.001	10.91	344.14

LMM with Random intercept and slope	4	IND	0.212	< 0.001	0.236	4.51	38.17
	
	5	AR1	0.209	< 0.001	0.234	5.90	42.93
	
	6	UNS	< 0.001	< 0.001	0.262	-0.57	339.39

SMA	7	-	0.273	< 0.001	0.241	-	-

Based on the results of the LMMs 4 and 5 selected on the basis of BIC, and also the SMA, one can accept that the rates of NO metabolites change in the three groups do not differ. Although this result coincides with that obtained by the most complicated model 6, the unsuitable models 1 and 3 reject the hypothesis of no interaction effect which is not illustrated in Figure 2.

All the LMMs, as well as the SMA, confirmed the effect of time on increasing the mean change over time in all of the groups. Except for the unreasonable model 6, all the models and the SMA confirmed that the mean change profiles for the three groups were the same throughout the time points; therefore, there was no significant group effect.

## Discussion

Based on the simulation and example results, it was found that obtaining accurate inferences in a LMM requires heavy statistical knowledge on the true and working covariance structures. However, due to developments in computer sciences, using mixed models is nowadays widespread in experimental designs and clinical trial studies where the sample sizes are not sufficiently large and/or sometimes the number of measurements is large. This serious aspect has previously been reported in a simulation study by Park [12] somewhat in a different way, where there was no random effect in the process of data generating. The interested reader is referred to [16-19] for the sample size and power calculations in repeated measurements analysis.

Interestingly, the SMA was robust to the true covariance structures in testing main and interaction effects even for small sample sizes and large number of measurements. Moreover, the SMA in the analysis of linear trend data was a powerful method in which its empirical powers were convincingly close to those of the best-fitting LMM, in general. This means that the least squares slope and mean of response are appropriate measures to summarize the corresponding effects.

In this study, we fitted the LMMs using the ''nlme'' package in the software R in which it follows the inner-outer approach for calculating the denominator degrees of freedom (df) of *F *statistics [20]. In comparison with the packages nlme and lme4 in R, the MIXED procedure in SAS provides also Satterthwaite and Kenward-Roger approximation methods for calculating the denominator df which especially result in some improvements in the resulting *p*-values. Although the superiority of these complex methods in terms of better preservation of type I error rates has been previously illustrated in unbalance designs [21-23], the differences are rather negligible when LMMs are employed inside the context of longitudinal analyses and there is no missing data. The R packages do have the advantage over the SAS procedure in providing the useful alternative algorithms Monte Carlo simulation and parametric bootstrap for getting more sensible *p*-values and confidence intervals. However, they are computationally intensive to be included in a simulation study.

The SMA clearly dominated the traditional UMA in testing time and interaction effects. The reason is that the SMA utilizes the linear trend in such data by computing the least squares slopes. However, the UMA assumes a more general nonlinear model with more parameters which must be estimated, and also imposes the most complex structure on the covariances of errors in which it may not be necessary.

Though not reported here some simulations based on the non-normal data show that, in general, the approaches were relatively robust to departures from multivariate normality. However, this had been reported previously for the two-sample Hotelling's *T^2 ^*test [24,25] and somewhat LME models [26,27].

This paper did not aim to deal with missing observations and baseline or pre-treatment measurement techniques. If the missing observations do not occur completely at random, it can introduce potential bias into parameter estimation and decision-making in statistical models. Barton and Cramer [28] and Catellier and Muller [29] have proposed several approximating denominator df on this issue. In this respect, the performance of SMA is highly dependent on weighting the individual's summary statistics [30] which may be cumbersome in practice. There are also more complex and efficient approaches to adjust the effect of baseline value (values) for the SMA such as including the baseline (average of baselines) or estimated intercept as covariate in an analysis of covariance (ANCOVA) model [4].

## Conclusions

It was shown that the SMA, on the basis of the two summary measures, was a simple, safe and powerful method in testing main and interaction effects in which it performed reasonably as the best-fitting LMM. However, The LMM often led to seriously inflated type I error rates and hence non-sensible inferences when the covariance structure for errors is misspecified. Moreover, this simple approach dominated the widely used UMA in assessing the linear trend data from a mixed model framework. The SMA is recommended as the first choice to confidently analyze linear trend data with a moderate to large number of measurements and/or small to moderate sample sizes.

## Competing interests

The authors declare that they have no competing interests.

## Authors' contributions

MV recommended the basics of the paper, carried out the simulations and prepared the majority of the manuscript. SMTA reviewed the literatures, guided the simulations and helped to draft the manuscript. MT participated in describing the simulation results and helped to draft the manuscript. FK provided the data of example 2, and participated in analyzing the dataset. All authors approved the final version of manuscript and did not have any competing interests.

## Pre-publication history

The pre-publication history for this paper can be accessed here:

http://www.biomedcentral.com/1471-2288/12/33/prepub
